# Segmental Resection With Primary Reconstruction Using a Patient-Specific Implant for Unicystic Ameloblastoma: A One-Year Follow-Up Case Report

**DOI:** 10.7759/cureus.40903

**Published:** 2023-06-24

**Authors:** Yashwanth V Satyanarayana Killampalli, Senthilnathan K P, Murugesan Krishnan, Melvin George, Sabari Nathan Rajamoorthy

**Affiliations:** 1 Oral and Maxillofacial Surgery, Saveetha Dental College and Hospital, Chennai, IND

**Keywords:** primary reconstruction, segmental resection, mural ameloblastoma, dental, quality of life (qol), patient specific implant (psi)

## Abstract

Unicystic ameloblastoma is a slow-growing tumor originating from the odontogenic epithelium that can be localized within the lining of a cyst. It commonly affects younger individuals and is frequently found in the posterior mandible. The classification of this tumor is based on histopathological characteristics, distinguishing between the luminal, intraluminal, and mural proliferation of the odontogenic epithelium. Treatment options vary depending on the histology and can range from enucleation to resection with secondary reconstruction. In recent years, patient-specific implants have gained popularity in reconstructive surgeries, particularly in craniomaxillofacial surgery. This case report focuses on a 22-year-old female patient with a mural-type unicystic ameloblastoma. The treatment involved segmental mandibular resection with primary reconstruction using a patient-specific implant to address the mandibular defect. The postoperative healing process and condylar movement were evaluated, and the patient achieved satisfactory results. This case report provides valuable insights into the management of primary reconstruction using a patient-specific implant.

## Introduction

Ameloblastoma is the second most common benign odontogenic tumor, following odontoma [1]. This tumor was first identified by Cusack in 1827 and is the most frequently occurring odontogenic tumor in the mandible [2]. It is a true neoplasm of enamel organ-type tissue that does not progress to the formation of enamel. The histopathological categorization of ameloblastoma has experienced revisions over time, with the latest update provided by the World Health Organization (WHO) in 2017. According to the current classification, ameloblastomas are grouped under benign epithelial odontogenic tumors, with further subdivisions including unicystic, peripheral, and metastasizing ameloblastomas. It is worth noting that the most recent edition of the WHO classification in 2022 now classifies unicystic ameloblastoma as a benign epithelial odontogenic tumor without a specific subclass [3]. Ameloblastoma can present as either a solid mass or a cyst-like structure, although it represents only 11% of all odontogenic tumors. While ameloblastomas typically grow slowly and are non-cancerous, they can occasionally exhibit aggressive behavior. Unicystic ameloblastoma differs significantly from the conventional type, appearing in a relatively younger age group, displaying a typically unilocular appearance on radiographs, and macroscopically resembling a cyst [4].

The treatment for unicystic ameloblastoma depends on the extent of the lesion and ranges from enucleation to resection with a free fibula flap and reconstruction plate [5]. In recent times, patient-specific implants have emerged as a valuable tool in reconstructive surgeries, reducing the complexity and associated morbidities. These implants are custom-made using computer-aided surgical simulation technology and can be designed to match the patient's anatomy, resulting in reduced operating time. Patient-specific implants have gained popularity in various reconstruction procedures, particularly in craniomaxillofacial surgery. They are classified based on the materials used, such as polymethylmethacrylate (PMMA), porous high-density polyethylene (pHDPE), and titanium mesh. Previous studies have demonstrated their clinical and surgical efficacy [6].

This case report presents the clinical presentation of a 22-year-old female patient with a mural-type unicystic ameloblastoma situated in the posterior region of the left mandible. The treatment protocol encompassed a segmental resection procedure, followed by primary reconstruction utilizing a patient-specific implant, all performed under general anesthesia. After one year from the completion of treatment, follow-up orthopantomogram images indicated discernible changes that imply alterations in the movement of the condylar unit within the glenoid fossa.

## Case presentation

A 22-year-old female presented to the Oral and Maxillofacial Surgery Department with the chief complaint of swelling on the lower left side of her face, which has been persistent for the last two weeks. The patient gives a history of swelling in the left mandibular region, which has been asymptomatic. The swelling has been noticeable for the past two weeks, although the patient is unable to recall its specific onset. There is no record of any trauma, pain, or dental procedure in that region. Furthermore, there is no relevant familial, medical, or dental history that could contribute to the condition.

Extra oral examination

**Figure 1:**
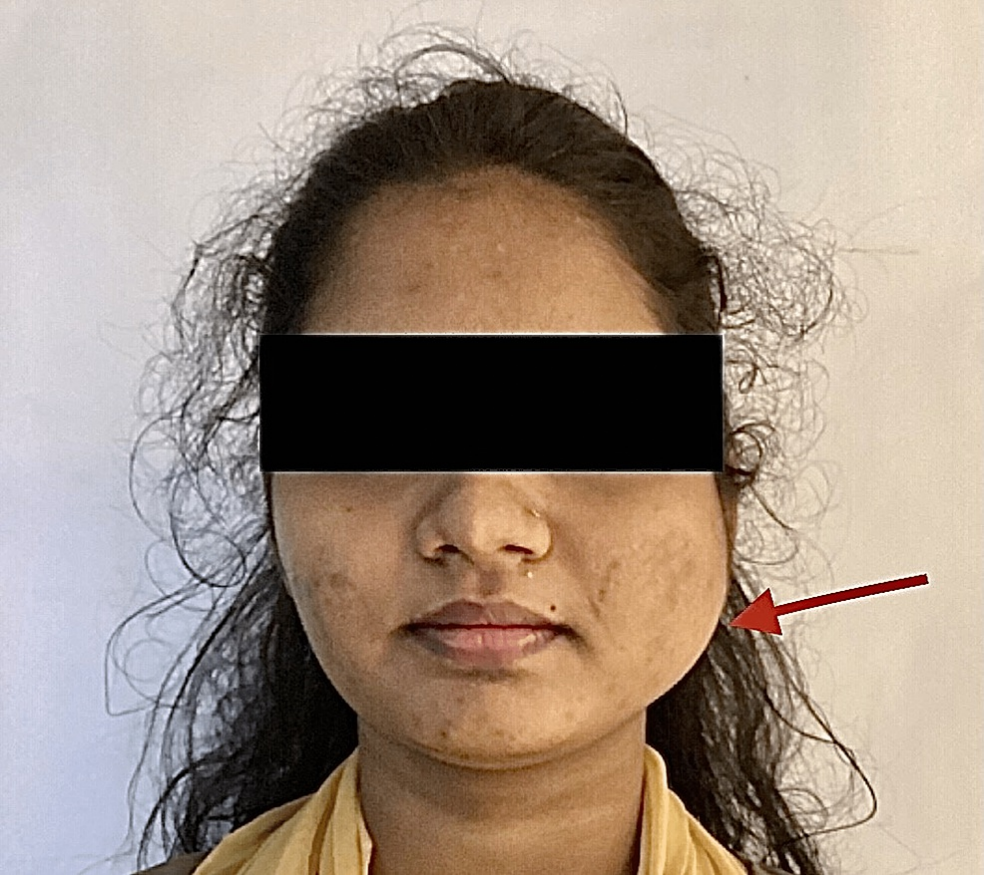
Preoperative image of the swelling The arrow represents the swelling in the left posterior mandibular region preoperatively

Upon inspection, a diffuse swelling measuring 3x4 cm was observed on the left posterior mandibular region. Anteroposteriorly, the swelling extended 3 cm from the left corner of the mouth to the angle of the mandible, while superoinferiorly, it extended from 1 cm below the tragus to the inferior border of the mandible (Figure 1). Palpation revealed a smooth surface and poorly defined edges of the swelling. No tenderness was noted during palpation. The overlying skin was unaffected, and no increase in local temperature was detected. Additionally, no palpable lymph nodes were observed. 

Intraoral examination 

There are no abnormalities that were detected. The mouth opening measured 42mm, which was normal.

Radiographic examination

**Figure 2:**
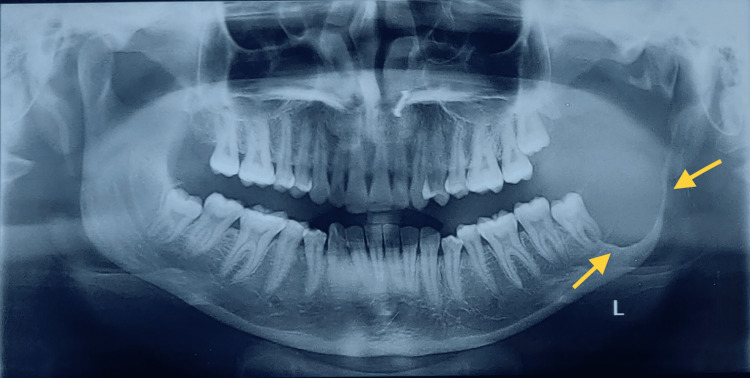
Radiograph showing the lesion in the left ramus of the mandible Arrows in the OPG show a well-defined lesion on the left Ramus of the Mandible involving a coronoid process extending towards the condyle.

The orthopantomographic radiograph (OPG) displayed a significant unicystic radiolucent lesion that covered a substantial portion of the left mandibular ramus. It extended from the sigmoid notch, the neck of the condyle, to the region of the left mandibular third molar (Figure 2). The presence of this lesion was accompanied by an impacted left third molar that was in close proximity to the distal root of the left second mandibular molar. The inferior alveolar nerve canal appeared to be displaced in an inferior direction. Based on the radiographic characteristics, as part of the differential diagnosis, the lesion exhibited similarities to odontogenic keratocysts, ameloblastomas, and dentigerous cysts. To differentiate between these conditions, it is necessary to consider their respective histopathological features.

**Figure 3:**
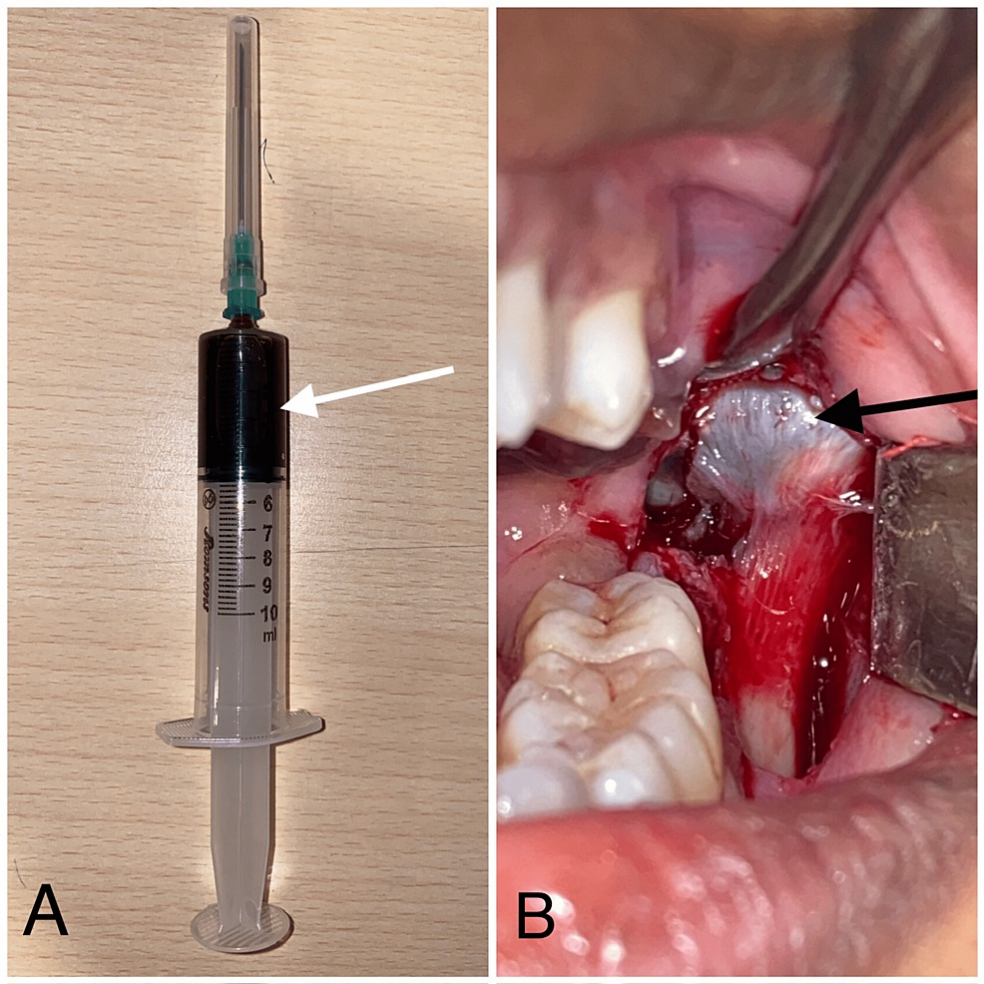
Needle aspiration and incisional biopsy The arrow in 3A shows a 10 ml syringe with serous fluid of a dark brown color, and the arrow in 3B shows a lesion during an incisional biopsy.

Following the examination, before the biopsy, a large 10 ml syringe with a 22-gauge needle was used to aspirate fluid from the site. The 44-aspirated fluid exhibited a serous consistency and a dark brown color, indicating disruption of the cystic lining or adjacent blood vessels, resulting in bleeding within the cystic space due to increased glandular activity. An incisional biopsy was then performed in the region of the third molar (Figure 3), with histopathology confirming the diagnosis of a mural variant of unicystic ameloblastoma.

**Figure 4:**
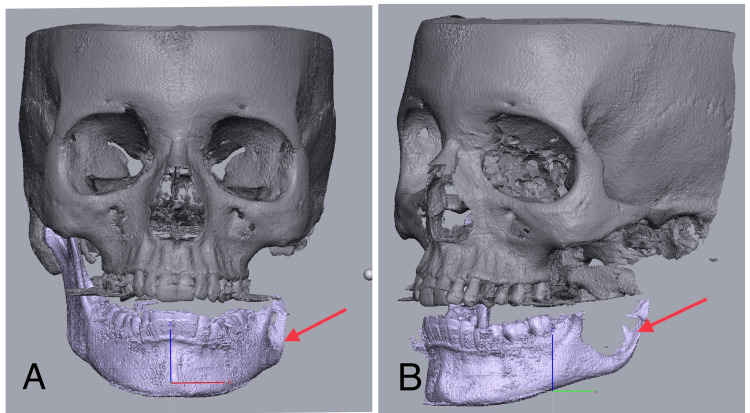
CBCT digital models using Geomagic Freeform software The arrow in 4A represents the lesion in frontal view and the arrow in 4B represents the lesion in lateral view. CBCT: Cone-beam computed tomography systems

A cone-beam computed tomography (CBCT) scan of the entire skull was performed to generate accurate digital models using Geomagic Freeform software (3D Systems, North Carolina, USA) (Figure 4).

**Figure 5:**
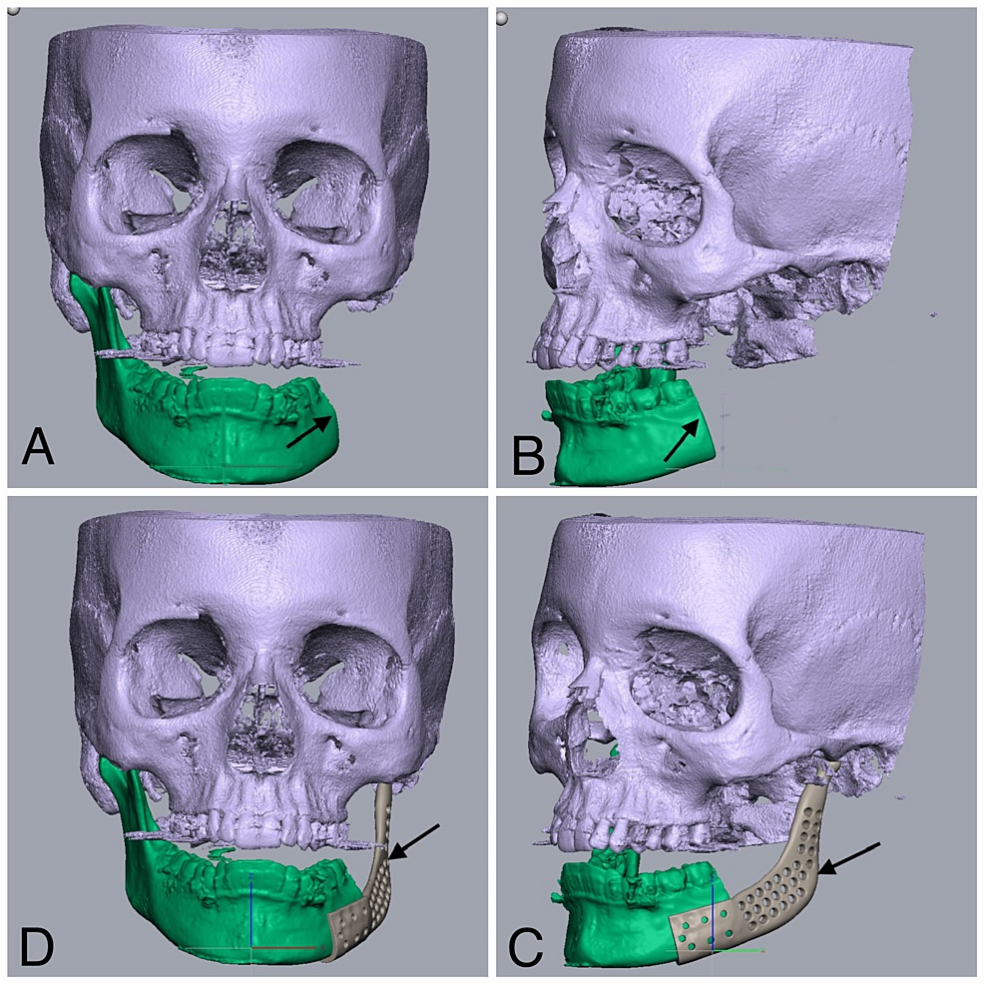
The CBCT digital models were utilized in Geomagic Freeform software to accurately position the osteotomy cuts and reconstruct a patient-specific implant The arrows in 5A and 5B represent the osteotomy cuts placed in the digital model, and the arrows in 5C and 5D represent the patient-specific implant reconstruction

Based on the findings from the clinical examination and three-dimensional CBCT measurements, the virtual planning of the osteotomy cut was carried out using Geomagic Freeform software. The osteotomy cut was positioned distal to the second molar in a perpendicular manner. Subsequently, a patient-specific implant was designed to match the specific requirements of the defect (Figure 5).

**Figure 6:**
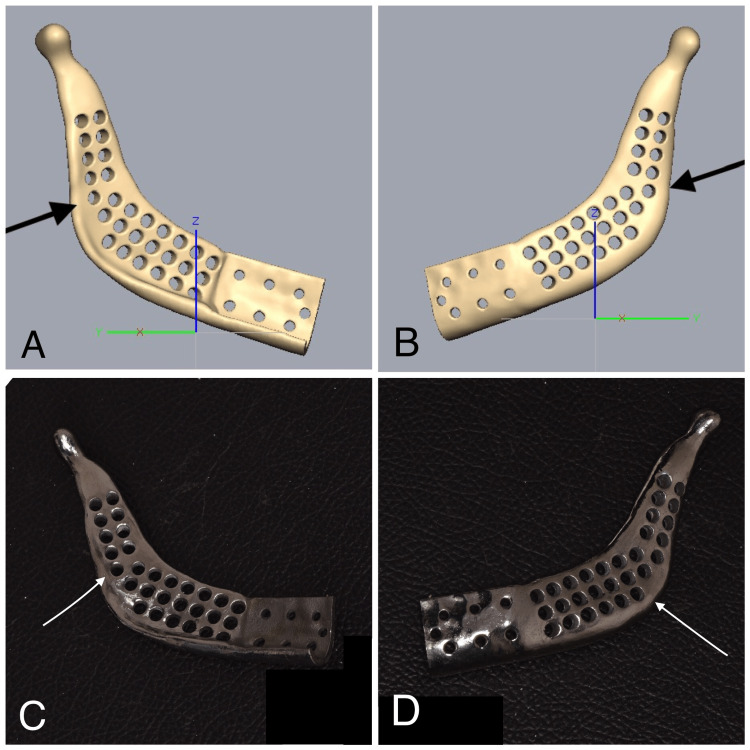
The digitally reconstructed patient-specific implant and the titanium-printed patient-specific implant The arrows in 6A and 6B represent the digitally reconstructed patient-specific implant, and the arrows in 6C and 6D represent the titanium-printed patient-specific implant.

Subsequently, the digital design was converted into a stereolithography file, which was then used to 3D print a patient-specific titanium implant (Figure 6).

**Figure 7:**
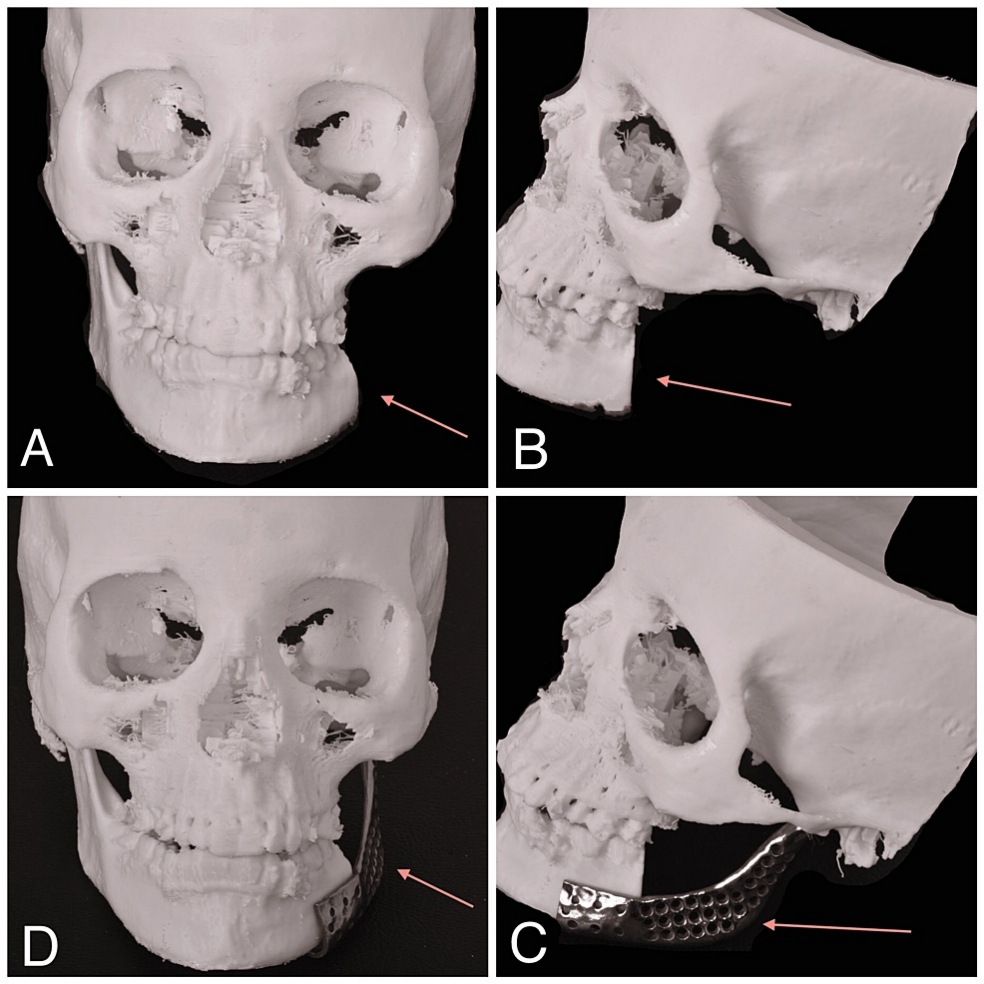
Stereolithographic model with a patient-specific implant The arrows in 7A and 7B represent the defective region in the stereolithographic model. The arrows in 7C and 7D represent the stereolithographic model adapted with a 3-D printed patient-specific implant

Following that, a full skull stereolithographic model with the defect was 3D printed to assess the fit of the patient-specific implant (Figure 7). Later, the patient-specific implant was double-sterilized for the surgical procedure.

Surgical procedure

**Figure 8:**
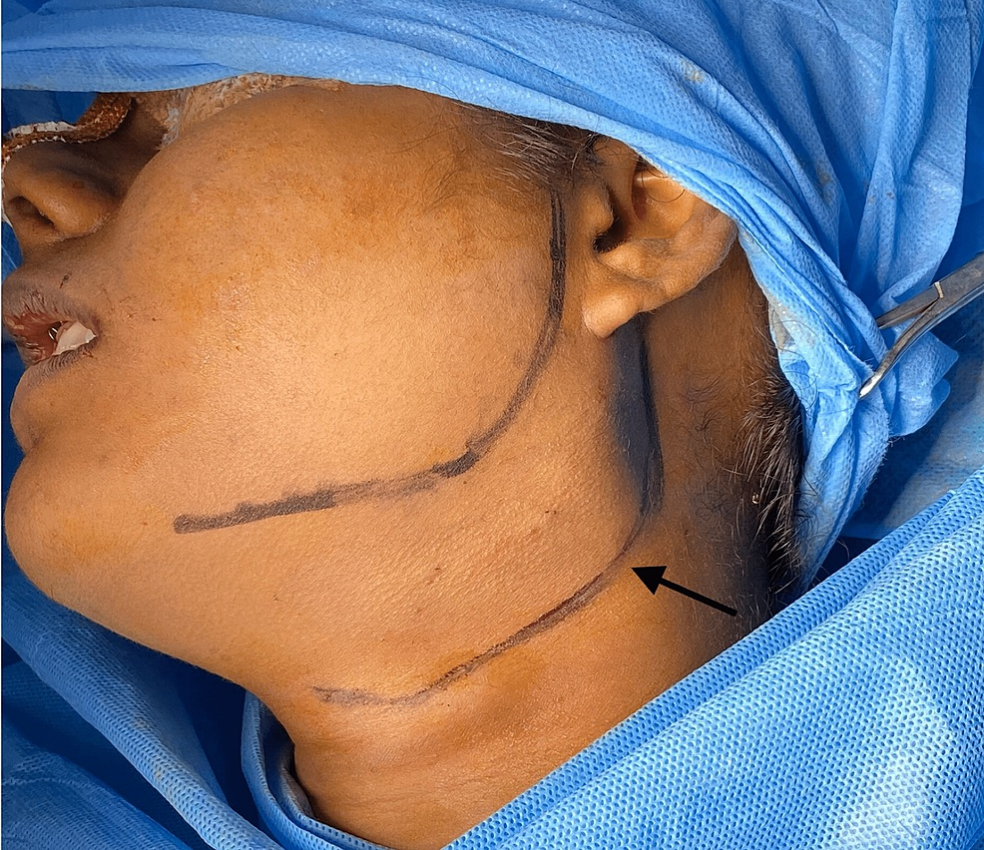
Submandibular incision marking The arrow in the image depicts the submandibular incision marking on the left side of the neck intraoperatively.

Under general anesthesia, naso-endotracheal intubation was performed using an endotracheal tube of size 7.0. standard scrubbing and draping protocols were followed. A Ryle's Tube was inserted and secured. A mixture of 2% Lignocaine diluted with adrenaline (1:200,000) was locally infiltrated around the soft tissue of the surgical site. A submandibular incision was made (Figure 8).

**Figure 9:**
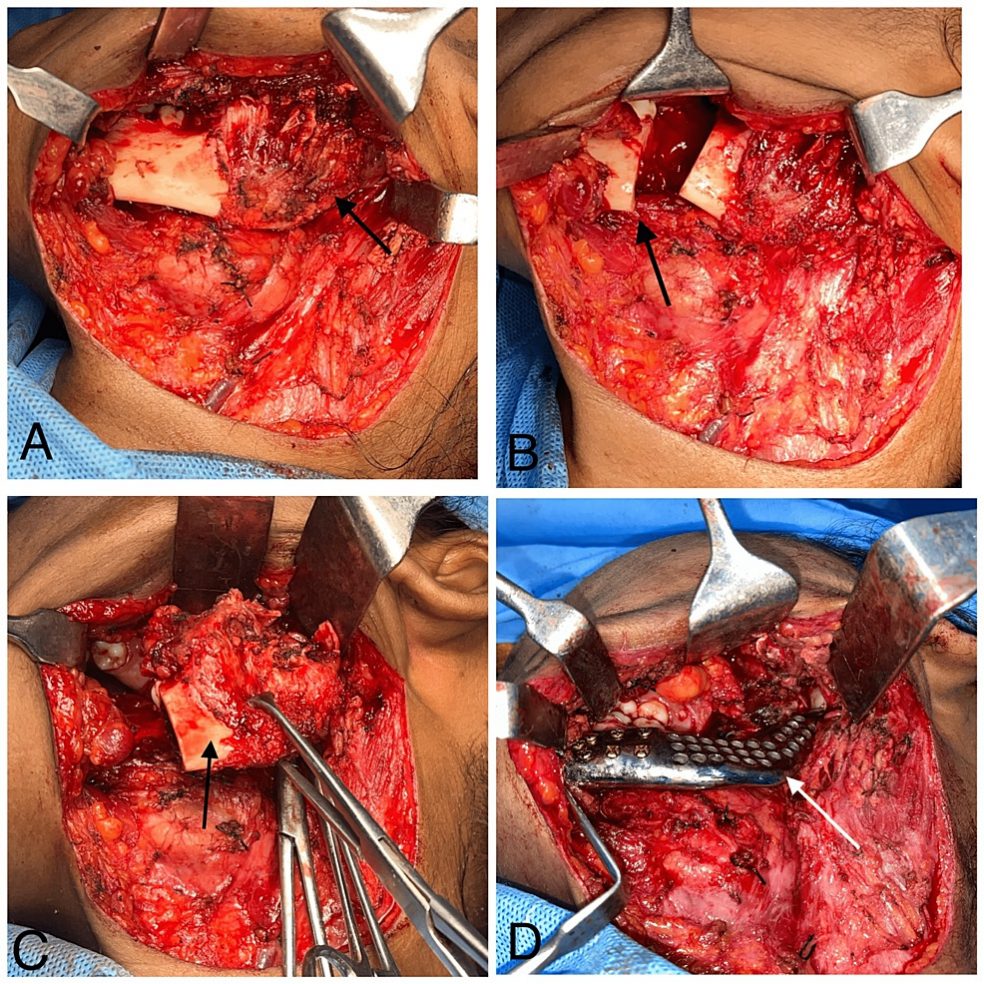
Intraoperative sequelae In Figure 9A, the arrow indicates the subplatysmal dissection performed and the subsequent raising of the flap. In Figure 9B, the arrow represents the segmental mandibulectomy conducted using an oscillating saw, precisely distal to the left mandibular second molar tooth, ensuring safe margins of 1cm. In Figure 9C, the arrow displays the excision of the lesion, as indicated by the arrow. Finally, in Figure 9D, the arrow signifies the fixation of the patient-specific implant.

Following subplatysmal dissection and flap raising (Figure 9A), the lower border of the mandible, cystic lining, coronoid process, and condylar process were identified through blunt dissection. An intraorally modified Ward incision extending along the ascending ramus was made. The flap was raised, and the cystic lining was identified. A segmental mandibulectomy was performed distal to the left second mandibular molar tooth with safe margins around 1 cm (Figure 9B). The lesion was excised (Figure 9C), with preservation of the condylar disc. A patient-specific implant was placed (Figure 9D), and fixation was achieved with a 2.5 mm profile, 12 mm, and 10 mm titanium screws. A neck drain was inserted and secured. Layer-wise suturing was performed using 3-0 Vicryl sutures and 2-0 Ethilon for primary closure.

Histopathological examination

**Figure 10:**
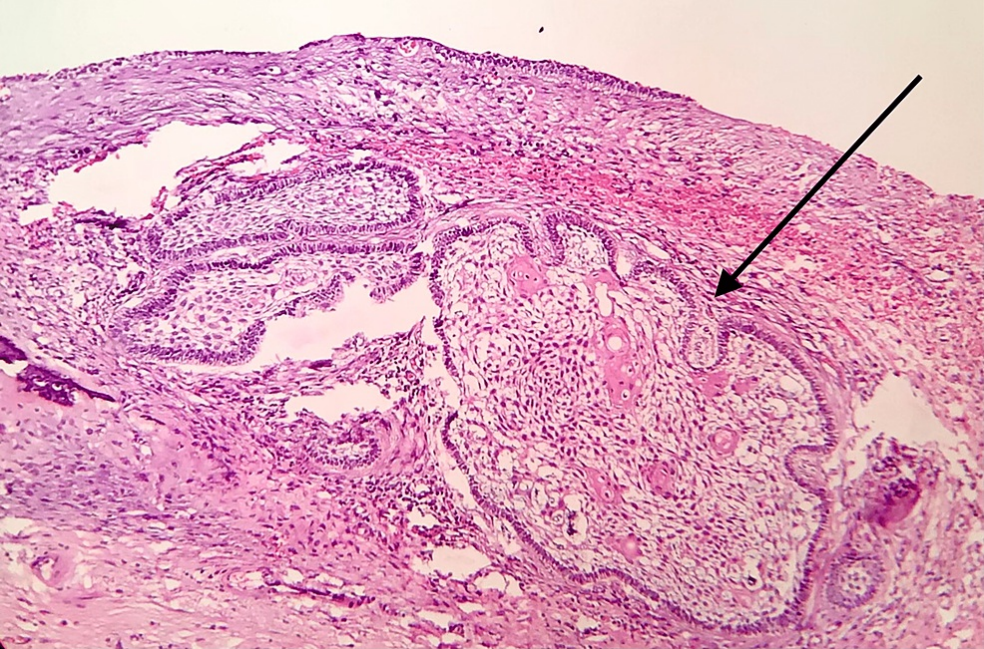
Hematoxylin and eosin stain photomicrographs are indicative of unicystic ameloblastoma with mural and intraluminal growth There is a presence of an odontogenic follicle (arrow), showing cystic degeneration and squamous metaplasia within the connective tissue wall.

The excised lesion was sent for histopathological examination. The examination revealed the presence of a cystic cavity lined by odontogenic epithelial and connective tissue walls. The odontogenic epithelial lining consisted of tall columnar basal cells exhibiting a reversal of polarity and subnuclear vacuolization, resembling ameloblast-like cells and superficial stellate reticulum-like cells. There was an intraluminal proliferation of the odontogenic epithelium. In a few areas, the connective tissue wall showed the presence of odontogenic follicles with cystic degeneration and squamous metaplasia (acanthomatous change). Based on histopathological features, it was diagnosed as unicystic ameloblastoma (mural and intraluminal variants) (Figure 10).

**Figure 11:**
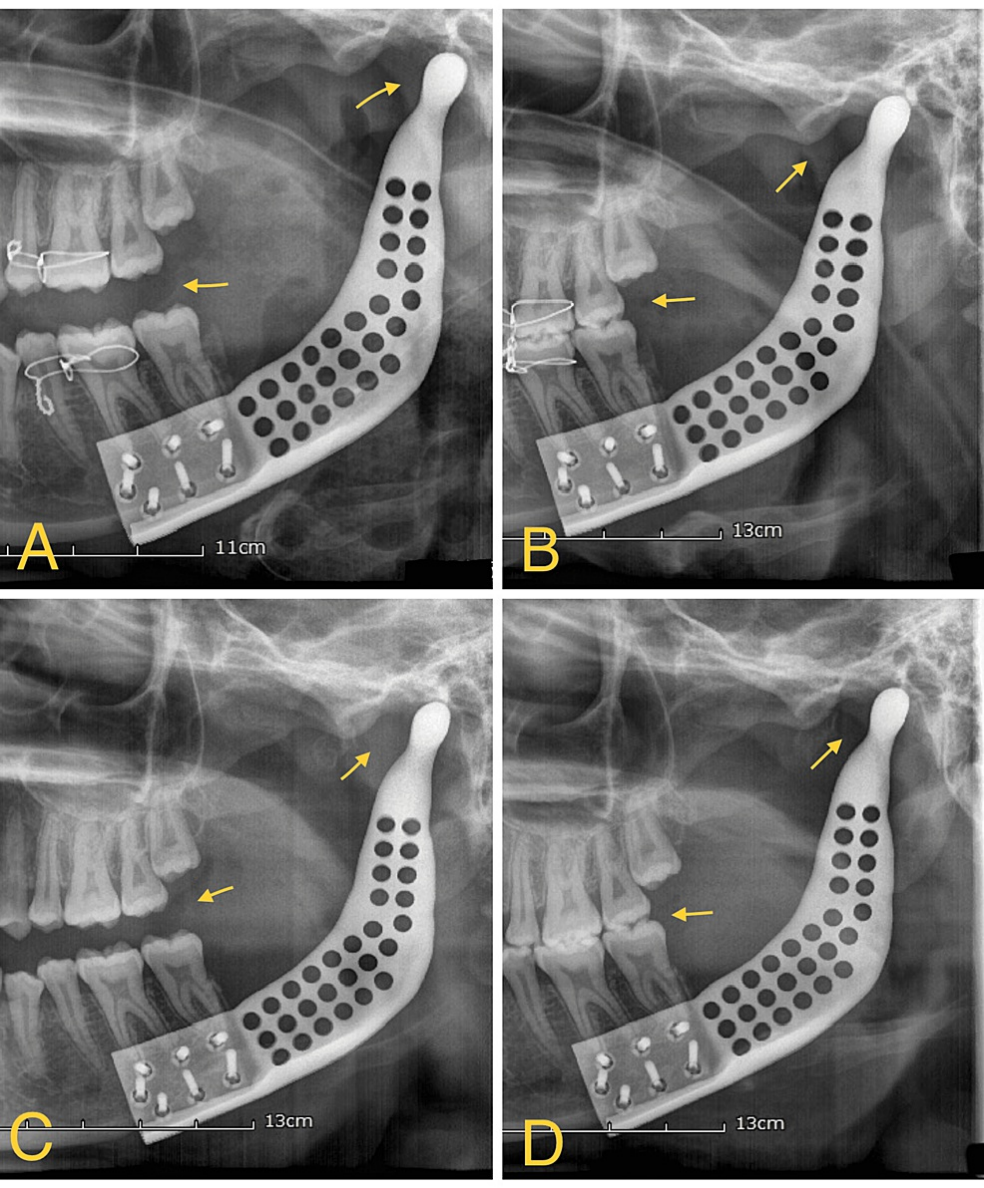
Post-operative condyle repositioning In Figure 11A, the arrow indicates the immediate postoperative period. The condyle was observed to be displaced from its normal position within the fossa. In Figure 11B, the arrow represents the restoration of the condyle to its proper position after intermaxillary fixation on the first postoperative day. Figure 11C ‘s arrow displays the condyle position after four months of operation. Finally, in Figure 11D, the arrow signifies the normal adaptation of the condyle after eight months postoperatively.

The postoperative healing process was uneventful. Subsequently, an orthopantomogram (OPG) was obtained to assess the position of the condyle and the occlusal relationship (Figure 11). During the immediate postoperative period, the condyle was observed to be displaced from its normal position within the fossa (Figure 11A). On the first postoperative day, intermaxillary fixation was performed, and OPG confirmed the restoration of the condyle to its proper position (Figure 11B). The patient was subsequently maintained on intermaxillary fixation for six weeks and received nutrition through a gastroenteric feeding tube. The intermaxillary fixation was later removed.

At the four-month mark, the patient reported pain in the temporomandibular joint (TMJ) during mouth opening. An orthopantomogram (OPG) was taken to reassess the joint space (Figure 11C). The patient was advised to consume soft foods and was prescribed Zerodol P for pain relief. Subsequently, the pain subsided. During the eighth month, a follow-up OPG was conducted to reevaluate the patient's condition (Figure 11D). The OPG revealed occlusal stability with good condyle adaptation to the fossa and pain reduction.

**Figure 12:**
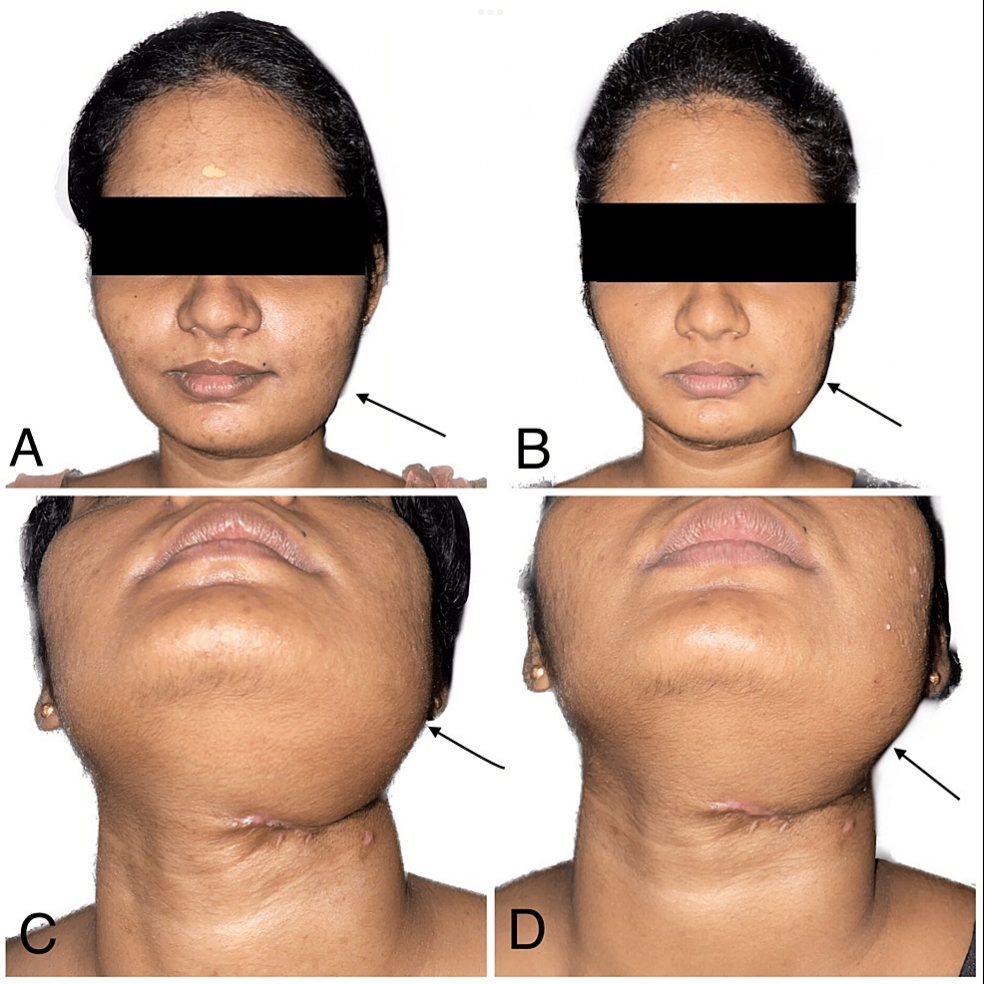
Facial swelling in the patient was noted at the fourth and eighth months In Figures 12A, 12C, arrows illustrate the presence of swelling observed during the fourth month. Conversely, In Figures 12B, 12D, the arrows illustrate the swelling observed during the eighth month, demonstrating a gradual reduction over time.

Facial swelling in the patient was noted in the fourth and eighth months, demonstrating a gradual reduction over time (Figure 12). 

**Figure 13:**
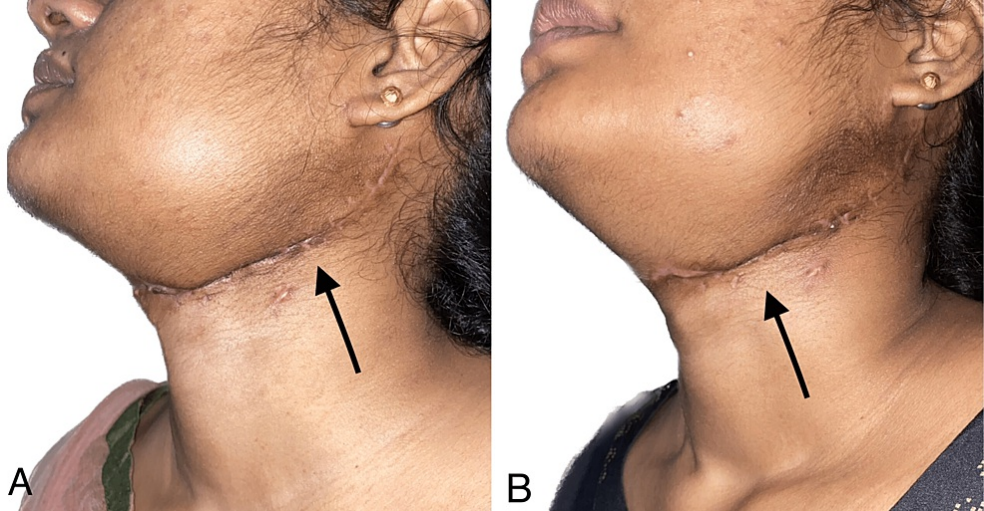
Neck scars noted at the fourth and eighth months In Figure 13A, the arrow displays the neck scar during the fourth month. Conversely, in Figure 13B, the arrow displays the neck scar during the eighth month

A neck scar in the patient was noted in the fourth and eighth months, demonstrating a gradual reduction over time (Figure 13).

## Discussion

In the case report, the effectiveness of patient-specific implants (PSIs) in reconstructing mandibular defects (Brown Class 1c), which are commonly associated with unicystic ameloblastoma development, as compared to traditional methods such as free fibula flap reconstruction [7]. Patient-specific implants offer several advantages, including reduced intraoperative time and donor-site morbidity. These implants are specifically designed based on the normal relationship between the condyle and glenoid fossa, which aids in restoring contour, achieving normal occlusion, and preserving joint function. In contrast, traditional methods have been associated with complications such as flap failure, infection, and donor site morbidity [8]. One notable advantage of using the Geomagic Freeform 3D Design and Sculpting Software is its ability to facilitate mock surgery planning, allowing visualization of postoperative changes. Additionally, the software enables the creation of stereolithographic models, which can be printed and assessed for proper fit with the patient-specific implant. Another advantage of the patient-specific implant is its lighter weight, as it is designed as a single unit, unlike the fibula bone with reconstruction plate commonly used in traditional methods [9]. In our case, the presence of odontogenic follicles within the connective tissue has led to a high recurrence rate in unicytic ameloblastoma with mural growth. Due to the significant likelihood of recurrence, we opted for segmental resection as the treatment approach. Studies have reported recurrence rates for ameloblastoma ranging from 15% to 25% after radical treatment and as high as 75% to 90% after conservative treatment [10]. Conservative surgical methods like enucleation and local curettage have demonstrated an unacceptably high recurrence rate [11]. In this case, regular monthly follow-ups were conducted. During the fourth month, the patient complained of pain while opening the mouth, prompting a reassessment of the joint space through an orthopantomogram (OPG) (Figure 11c). Zerodol P was prescribed for 10 days, which resulted in a subsequent reduction in pain. By the eighth month, the patient had no fresh complaints, and the OPG revealed normal occlusion, joint relationship, and facial symmetry (Figure 11d). In a similar study by Olate et al. [12], computer-aided design and computer-aided manufacturing (CAD-CAM) patient-specific implants (PSIs) were used for Temporomandibular Joint Replacement in patients with end-stage TMJ arthritis. They concluded that the development of a TMJ prosthesis resulted in the absence of pain and the restoration of normal mouth opening after 12 months of follow-up, along with improved facial symmetry.

Mukul SK et al. [13] presented cases of facial trauma resulting in secondary deformities due to left zygomatico-orbital-maxillary complex fractures. The patients underwent zygomatic osteotomies, followed by reconstruction of the left zygoma and orbital floor using patient-specific implants (PSIs) made of titanium. The authors concluded that PSIs offer an accurate and efficient alternative for craniomaxillofacial defect reconstruction, yielding satisfactory aesthetic results, minimizing postoperative complications, and reducing the risks associated with autogenous grafts. However, the high cost of PSIs remains a drawback despite their advantages in terms of safety and time efficiency due to preoperative digital planning and fabrication compared to traditional methods. Moreover, once the PSIs are fabricated, changes in the treatment plan are not possible during surgery, as these implants cannot be bent or reshaped. Additionally, foreign materials like PSIs pose a significant risk of infection.

Similarly, Alasseri N. et al. [14] successfully reconstructed various maxillofacial defects in six patients using patient-specific implants. They utilized a total of 10 implants, with eight fabricated from polyetheretherketone (PEEK) and two from titanium. No postoperative complications were observed. Another case report by Hurrell MJL et al. [15] demonstrated the successful preservation of the native temporomandibular joint (TMJ) in two cases with a very short salvageable condylar component. This was achieved using 3D-printed patient-specific cutting guides and implants. The technique described effectively preserved the TMJ capsule and intra-articular components, maintaining their relationships, and resulted in excellent outcomes for both patients. This preservation approach offers a higher likelihood of achieving normal occlusion, segment alignment, and condylar translation while minimizing potential complications. However, in our case report, complete disarticulation of the condyle was performed, and yet satisfactory occlusion was achieved.

This case report presents a novel approach to segmental mandibulectomy by utilizing a patient-specific implant for immediate reconstruction. Previously, a free fibula flap with titanium reconstruction plates and condylar units was commonly used for treating such tumors. Understanding appropriate biopsy techniques is crucial for accurate diagnosis and treatment planning. The possibility of primary reconstruction improves the patient's quality of life by avoiding the need for a second surgery. However, comparative studies between patient-specific implants and traditional methods are necessary, and further case reports and series should be published to facilitate knowledge sharing among surgeons and pathologists.

## Conclusions

To date, there have been limited reports in the literature regarding primary reconstruction with patient-specific implants after segmental resection for unicystic ameloblastoma. Limited cases specifically addressed patient-specific condyle reconstruction during the initial surgical resection. This report introduces a novel technique whereby patient-specific implants are employed as the primary method of reconstruction following segmental resection. Additionally, it addresses the management of reconstruction in the patient-specific condyle unit, emphasizing the preservation of the condylar disc and the achievement of successful outcomes without the necessity of a fibula flap. This case emphasizes the value of digital planning in reconstructing bony defects of the jaws, leading to successful outcomes.

## References

[1] Pieter J S (2019). Jaws cancer: pathology and genetics. Encyc Can.

[2] Ghai S (2022). Ameloblastoma: an updated narrative review of an enigmatic tumor. Cureus.

[3] Soluk-Tekkesin M, Wright JM (2022). The World Health Organization classification of odontogenic lesions: a summary of the changes of the 2022 (5th) edition. Turk Patoloji Derg.

[4] Pradeep Pradeep (2021). Unicystic mural ameloblastoma: an case report and review of literature. Int J Dent Oral Sci.

[5] Chaudhary Z, Sangwan V, Pal US, Sharma P (2011). Unicystic ameloblastoma: a diagnostic dilemma. Natl J Maxillofac Surg.

[6] Thayaparan GK, Lewis PM, Thompson RG, D'Urso PS (2021). Patient-specific implants for craniomaxillofacial surgery: A manufacturer's experience. Ann Med Surg (Lond).

[7] Ragbir M, Brown JS, Mehanna H (2016). Reconstructive considerations in head and neck surgical oncology: United Kingdom National Multidisciplinary Guidelines. J Laryngol Otol.

[8] EK Chee (2007). Fibula osteocutaneous flap for mandible reconstruction after ameloblastoma resection: amending technique to reduce ischaemic time. Mala Ortho Jr.

[9] Huang MF, Alfi D, Alfi J, Huang AT (2019). The use of patient-specific implants in oral and maxillofacial surgery. Oral Maxillofac Surg Clin North Am.

[10] Infante-Cossio P, Prats-Golczer V, Gonzalez-Perez LM (2013). Treatment of recurrent mandibular ameloblastoma. Exp Ther Med.

[11] Dandriyal R, Gupta A, Pant S, Baweja HH (2011). Surgical management of ameloblastoma: conservative or radical approach. Natl J Maxillofac Surg.

[12] Olate S, Bahls V, Uribe F, Unibazo A, Martínez F (2020). Patient-specific implant for temporomandibular joint replacement in juvenile arthritis and facial asymmetry. Ann Maxillofac Surg.

[13] Mukul SK, Mishra M, Singh G (2019). Patient-specific implants in maxillofacial reconstruction: a case report. Traumaxilla.

[14] Alasseri N, Alasraj A (2020). Patient-specific implants for maxillofacial defects: challenges and solutions. Maxillofac Plast Reconstr Surg.

[15] Hurrell MJ, Singh J, Leinkram D, Clark JR (2022). Patient specific implant with high condylar neck osteotomy for temporomandibular joint preservation in segmental mandibulectomy. Oral Oncol.

